# Roswell Park, A. M., M. D.

**Published:** 1893-02

**Authors:** Ferd. C. Valentine

**Affiliations:** New York


### Correspondence.

NEW YORK, January 3, 1893.

*Roswell Park, A. M., M. D., 510 Delaware avenue, Buffalo, N. Y.:*

DEAR DR. PARK—One-thirteenth part of your interesting paper on Actinomycosis, read before the Buffalo Academy of Medicine, and published in the BUFFALO MEDICAL AND SURGICAL JOURNAL for January, 1893, is a copy, *verbatim, literatim et punctatim*, from *Condensed Extracts* for August, 1892.

Your own work cannot leave you unaware of the vast amount of labor the preparation of such matter entails. The free hand with which authors and journals avail themselves of my arduous search among over 400 medical journals monthly, without even giving me credit, oblige me to protect myself.

If the next issue of the BUFFALO MEDICAL AND SURGICAL JOURNAL contains a statement, over your signature, acknowledging that one of the thirteen pages of your article was taken bodily from *Condensed Extracts*, thus demonstrating your appreciation of my little journal as an aid to medical authors, specialists, and general practitioners, that *Condensed Extracts* represents about $1,600 worth of annual subscriptions to foreign medical journals, which I furnish at $1 per year, and that *Condensed Extracts* is published at 256 West Fifty-first street, New York, whither you advise all to send for a sample copy, which, in your opinion, will lead to becoming regular subscribers, then I will deem that you did not intentionally violate the copyright which protects every line in *Condensed Extracts.*

Failing in the above, I shall be obliged to take such further steps as I may be advised.

Looking to you for justice, I am, dear Doctor,

Yours very truly,

FERD. C. VALENTINE.

